# Evaluation of the Needs and Experiences of Patients with Hypertriglyceridemia: Social Media Listening Infosurveillance Study

**DOI:** 10.2196/44610

**Published:** 2023-12-19

**Authors:** Junxian Song, Yuxia Cui, Jing Song, Chongyou Lee, Manyan Wu, Hong Chen

**Affiliations:** 1 Beijing Key Laboratory of Early Prediction and Intervention of Acute Myocardial Infarction, Department of Cardiology Center for Cardiovascular Translational Research Peking University People’s Hospital Beijing China

**Keywords:** social media listening, hypertriglyceridemia, infosurveillance study, disease cognition, lifestyle intervention, lipid disorder, awareness, online search, telemedicine, self-medication, Chinese medicine, natural language processing, cardiovascular disease, stroke, online platform, self-management, Q&A search platform, social media

## Abstract

**Background:**

Hypertriglyceridemia is a risk factor for cardiovascular diseases. Internet usage in China is increasing, giving rise to large-scale data sources, especially to access, disseminate, and discuss medical information. Social media listening (SML) is a new approach to analyze and monitor online discussions related to various health-related topics in diverse diseases, which can generate insights into users’ experiences and expectations. However, to date, no studies have evaluated the utility of SML to understand patients’ cognizance and expectations pertaining to the management of hypertriglyceridemia.

**Objective:**

The aim of this study was to utilize SML to explore the disease cognition level of patients with hypertriglyceridemia, choice of intervention measures, and the status quo of online consultations and question-and-answer (Q&A) search platforms.

**Methods:**

An infosurveillance study was conducted wherein a disease-specific comprehensive search was performed between 2004 and 2020 in Q&A search and online consultation platforms. Predefined single and combined keywords related to hypertriglyceridemia were used in the search, including disease, symptoms, diagnosis, and treatment indicators; lifestyle interventions; and therapeutic agents. The search output was aggregated using an aggregator tool and evaluated.

**Results:**

Disease-specific consultation data (n=69,845) and corresponding response data (n=111,763) were analyzed from 20 data sources (6 Q&A search platforms and 14 online consultation platforms). Doctors from inland areas had relatively high voice volumes and appear to exert a substantial influence on these platforms. Patients with hypertriglyceridemia engaging on the internet have an average level of cognition about the disease and its intervention measures. However, a strong demand for the concept of the disease and “how to treat it” was observed. More emphasis on the persistence of the disease and the safety of medications was observed. Young patients have a lower willingness for drug interventions, whereas patients with severe hypertriglyceridemia have a clearer intention to use drug intervention and few patients have a strong willingness for the use of traditional Chinese medicine.

**Conclusions:**

Findings from this disease-specific SML study revealed that patients with hypertriglyceridemia in China actively seek information from both online Q&A search and consultation platforms. However, the integrity of internet doctors’ suggestions on lifestyle interventions and the accuracy of drug intervention recommendations still need to be improved. Further, a combined prospective qualitative study with SML is required for added rigor and confirmation of the relevance of the findings.

## Introduction

Hypertriglyceridemia is a common lipid disorder that may lead to cardiovascular diseases with a worldwide prevalence of 10% in the adult population [1,2]. The complex etiologic nature of hypertriglyceridemia makes diagnosis and management challenging to physicians in clinical practice, requiring intervention with lipid-lowering medications along with lifestyle and dietary changes [3]. Recently, the China National Stroke Screening and Prevention Project reported a 22.4% and 22.5% prevalence of hypertriglyceridemia among Chinese adults of the rural and urban population, respectively [4]. Another recent survey on internet usage in China reported that the number of internet users in China reached 904 million with an internet penetration rate of 64.5% as of March 2020 [5]. This intensification of internet usage has resulted in a paradigm shift in the exchange of health care knowledge, with internet-based social media being one of the most important sources of health care information [6].

Social media hold considerable potential value in the health care system by offering versatile, online platforms for individuals with specific health conditions to map their symptoms to a specific disease condition. This will facilitate the self-management of their conditions, help to identify and access health care resources, enable networking with fellow patients, and communicate problems and experiences online [7,8]. Furthermore, social media platforms, especially disease-specific platforms (ie, discussion boards, patient forums, and question-and-answer [Q&A] platforms), provide a window to assess patients’ perceptions of their diseases, preferences and rationale for treatment decisions, outcomes, and other factors affecting their lives [9].

Using conventional methods (eg, patient questionnaires in different research settings and electronic medical records) for assessing the patient experience is challenging and subject to bias as these methods are tedious and troublesome [9,10]. Social media listening (SML) has emerged as a new approach to analyze and monitor online discussions related to various health-related topics in the context of diverse diseases to generate insights into users’ experiences and expectations. SML aids in mining and gauging the unmet needs of patients and doctors and provides innovative insights for pharmaceutical/medical industries [9].

To date, only a few studies have utilized SML to investigate various health-related issues in diverse therapeutic areas, including inflammatory bowel disease, cancer, chronic obstructive pulmonary disease, and dry-eye disease [7,9,11,12]. To our knowledge, only two studies have utilized SML to passively collect data from the Chinese population, including (1) prediction of the regional prevalence of diabetes, hypertension, and BMI for public health monitoring [13] and (2) to understand patients’ unmet needs to optimize communication between doctors and patients with Parkinson disease [14]. In 2018, the US Food and Drug Administration made a recommendation and encouraged the use of SML to shed light on unmet needs of doctors and patients [15]. However, no study has yet examined the utility of SML in the context of hypertriglyceridemia to understand patients’ cognizance and expectations pertaining to its management. Therefore, the aim of this study was to analyze the perception of patients with hypertriglyceridemia, choice of intervention measures, and status quo of online consultations using SML as the research tool.

## Methods

### Study Design and Data Source

This was an SML infosurveillance study designed to assess the overview of hypertriglyceridemia, disease perception in patients, intervention measures recommended by doctors (online consultation) and preferred by patients, and demographic characteristics of patients with hypertriglyceridemia. Data were extracted from six disease-specific Q&A search platforms and 14 online consultation forums in China related to hypertriglyceridemia (Table 1).

**Table 1:** Data source platforms.

Platforms		Data acquisition method
**Question & answer search**		
	Baidu Zhidao [16]	Keyword search
	Sogou Wenwen [17]	Keyword search
	360 Q&A [18]	Keyword search
	Iask [19]	Keyword search
	Baidu Tieba (Hyperlipidemia Tieba) [20]	Keyword search (only topic posts)
	Zhihu [21]	Keyword search (only question & answer topic posts)
**Online consultation^a^**		
	120 Ask [22]	Keyword search
	Xunyiwenyao (question and answer) [23]	Keyword search
	99 Health [24]	Keyword search
	WeDoctor Guahao (need to log in) [25]	Keyword search
	Qiuyi [26]	Keyword search
	Family Doctor (Instant ask and answer) [27]	Keyword search
	Dingxiang Doctor [28]	Keyword search
	Haodaifu [29]	Keyword search
	39 Ask [30]	Keyword search
	Feihua Health (question and answer) [31]	Keyword search
	Chinakang [32]	Keyword search
	99 Ask Doctor [33]	Keyword search
	Chunyu Yisheng [34]	Keyword search
	Baixing Wen Yisheng [35]	Keyword retrieval after full access to page data
	China Heart Health (stopped updating after 2014)	Keyword search

^a^In the consultative platforms, it is a general requirement to state all attributes of age, gender, disease status, and medical information.

### Search Strategy

A comprehensive search was performed between 2004 to 2020 using the following predefined keywords from the selected platforms: hypertriglyceridemia, hypertriglyceridemia and mixed hyperlipidemia, triglycerides, fenofibrate, lipanthyl, bezafibrate. Further, the keywords related to hypertriglyceridemia were combined with other search strings related to the disease, symptoms, diagnosis and treatment indicators, lifestyle interventions, and therapeutic agents using Boolean operators such as AND and OR (Table 2). The resulting search output was aggregated using a web crawler tool and evaluated.

**Table 2:** Search strategy keywords.

Search strategy			Keywords^a^
**Search by single keyword**			
	I-a	Hypertriglyceridemia, high TG, and mixed hyperlipidemia	
	II-a	Triglycerides, triacylglycerols, triglycerides, TG, triglycerides	
	IV-a	Bate, fenofibrate, lipanthyl, bezafibrate	
**Search by keyword combination (pairwise sum)**			
	I-a+II-a	(hypertriglyceridemia, high TG, mixed hyperlipidemia)+(triglycerides, triacylglycerol, TG, triglycerides)	
	I-a+II-b	(hypertriglyceridemia, high TG, mixed hyperlipidemia)+(blood lipid)	
	I-a+III	(hypertriglyceridemia, high TG, mixed hyperlipidemia)+(diet, food)	
	I-a+IV-a	(hypertriglyceridemia, high TG, mixed hyperlipidemia)+(bate, fenofibrate, lipanthyl, bezafibrate)	
	I-a+IV-b	(hypertriglyceridemia, high TG, mixed hyperlipidemia)+(nonbeta drugs)	
	II-a+I-b	(triglycerides, triacylglycerols, triglycerides, TG, triglycerides)+(hyperlipidemia, dyslipidemia, hyperlipidemia, thick blood lipids, three high^b^)	
	II-a+III	(triglycerides, triacylglycerols, triglycerides, TG, triglycerides)+(diet, food)	
	II-a+IV-a	(triglycerides, triacylglycerol, triglycerides, TG, triglycerides)+(bate, fenofibrate, lipanthyl, bezafibrate)	
	II-a+IV-b	(triglycerides, triacylglycerols, triglycerides, TG, triglycerides)+(non-beta drugs)	
	IV-a+I-b	(beta, fenofibrate, lipanthyl, bezafibrate)+(hyperlipidemia, dyslipidemia, hyperlipidemia, thick blood lipids, three high)	
	IV-a+II-b	(beta, fenofibrate, lipanthyl, bezafibrate)+(blood lipid)	

^a^Some terms repeated in English correspond to various terms for the same English word in Chinese: 甘油三酯, 三酸甘油脂, and 甘油三脂 all translate to “triglycerides”; 高脂血症 and 高血脂 both translate to hyperlipidemia.

^b^“three high” refers to a Chinese term for high blood pressure, hyperlipidemia, and hyperglycemia.

### Data Set Construction and Retrieval Path

A schematic illustration of the data retrieval path is provided in Multimedia Appendix 1. First, the Spider web crawler tool was used to obtain data from specified data sources based on different keyword/search string retrieval strategies. Second, the obtained data were subjected to preliminary data cleaning to remove advertisements, followed by deduplication to obtain analyzable data. Based on the data processing stage, the data obtained would fall under (1) original/source data, (2) valid data after data cleaning, and (3) data included in the analysis. It is expected that the quantity of data at each of these steps would vary and narrow down to obtain the data set finally included for the analysis. The attributes were manually defined and the standards were set by professionals, including experts in data management and authors who are cardiovascular disease specialists. Based on the set standards, data engineers conducted sampling, data annotation, and contextualizing of the data set. Subsequently, algorithm engineers conducted machine learning for data acquisition, sorting, and dimension extraction/prediction based on the corresponding rules. This step was followed by manual review and quality control/assurance performed by the data management professionals.

### Inclusion and Exclusion Criteria

Patients were included for analysis if they had an abnormal triglyceride (TG) level (≥1.7 mmol/L) with or without symptoms, abnormal TG levels with secondary diseases (cardiovascular disease and acute pancreatitis), or suspected hypertriglyceridemia who asked questions about an abnormal increase of TG and expressed specific concerns or knowledge about the disease. Data obtained from various sources were considered for analysis as per predefined inclusion and exclusion criteria (see Table 3 and Table 4, respectively). The keywords were manually customized for each set of inclusion criteria specific for each of the research objectives manually by the data management professionals in collaboration with the authors.

**Table 3:** Inclusion criteria.

Classification of inclusion rules		Feature keywords^a^
**Inclusion rule I (disease)**		
	I-a	Hypertriglyceridemia, high TG, and mixed hyperlipidemia
	I-b	Hyperlipidemia, dyslipidemia, thick blood lipids, three high^b^
**Inclusion rule II (diagnosis and treatment indicators)**		
	II-a	Triglycerides, triacylglycerols, triglycerides, TG, triglycerides
	II-b	Blood lipids
Inclusion rule III (lifestyle intervention)		Diet, food
**Inclusion rule IV (therapeutic drugs)**		
	IV-a	Bate, fenofibrate, lipanthyl, bezafibrate
	IV-b	Statins (statins), lipid-lowering drugs (hyperlipidemic drugs); lovastatin, simvastatin, pravastatin, fluvastatin, atorvastatin, rosuvastatin and pitavastatin; niacin, ezetimibe, probucol, fish oil, Xuezhikang, Zhibituo, Zhikening

^a^Some terms repeated in English correspond to various terms for the same English word in Chinese: 甘油三酯, 三酸甘油脂, and 甘油三脂 all translate to “triglycerides”; 高脂血症 and 高血脂 both translate to hyperlipidemia.

^b^“three high” refers to a Chinese term for high blood pressure, hyperlipidemia, and hyperglycemia.

**Table 4:** Data exclusion criteria.

Elimination rule	Elimination definition	Elimination method
Nontopic-related content	Triglycerides were low, triglycerides were relatively low, triglycerides were not high; the detection value of triglycerides was lower than 1.7 mmol/L	BERT^a^ text classification model: manual output of training set, train model, verification by manual training verification set, elimination of residual data by model
Duplicate ID	Duplicate page ID content	Compare ID characters and reject duplicate IDs
Advertising content	No description of patient’s personal illness, introduction of medical institutions, products, advertising links, invitation to join the group or join the consultation, the questioner is the organization	Text recognition article with jump links, link address for advertising, delete or manually output the training set, and use event sequence template mining to build the model for recognition
Popular science articles	Popular medical science, no description of patient’s condition	Mining of event sequence template using the BERT text classification model: manual output of training set, train model, verification by manual training verification set, elimination of residual data by model

^a^BERT: bidirectional encoder representations from transformers.

### Analysis Dimensions

A schematic illustration of the analysis dimensions for this study is provided in Multimedia Appendix 2. Descriptive analysis of the patient/user demographic details was carried out using deidentified and aggregated data. The age, gender, and other data attributes were identified using both machine learning and manual curation by trained professionals based on predefined criteria. Gender was assessed by either direct expressions or by attributes, including gender-associated suffixes/prefixes or adjectives/verbs, searched and correlated with the patient for prediction of gender in differentiating the web user or patient with a query either for self or on behalf of the patient (eg, the text “my father” was used to assign the gender of the patient as “male”). The age attributes were searched for both direct or indirect expressions and predicted based on the set criteria. A web user’s declaration of the patient’s age or description relating to the patient’s age by an indirect method was categorized under the set range. The major study objective was to provide an overview on hypertriglyceridemia, patient cognitive status, intervention selection, and an analysis of intervention suggestions from online consultations and Q&A search platforms.

### Ethical Considerations

The study was approved by the Ethics Committee of Peking University People’s Hospital (2020PHB096). All data were obtained from publicly available sources and have been aggregated and anonymized. Only the deidentified data without personal identification were used for analysis. This study did not directly involve human participants nor did it include any intervention. Additionally, no data pertaining to users’ personally identifiable information are presented in the text or figures of this article.

### Statistical Analysis

Data obtained were analyzed using descriptive statistics. Categorical variables are presented as frequencies and percentages. 

## Results

### Overview of Data

**Figure 1:**
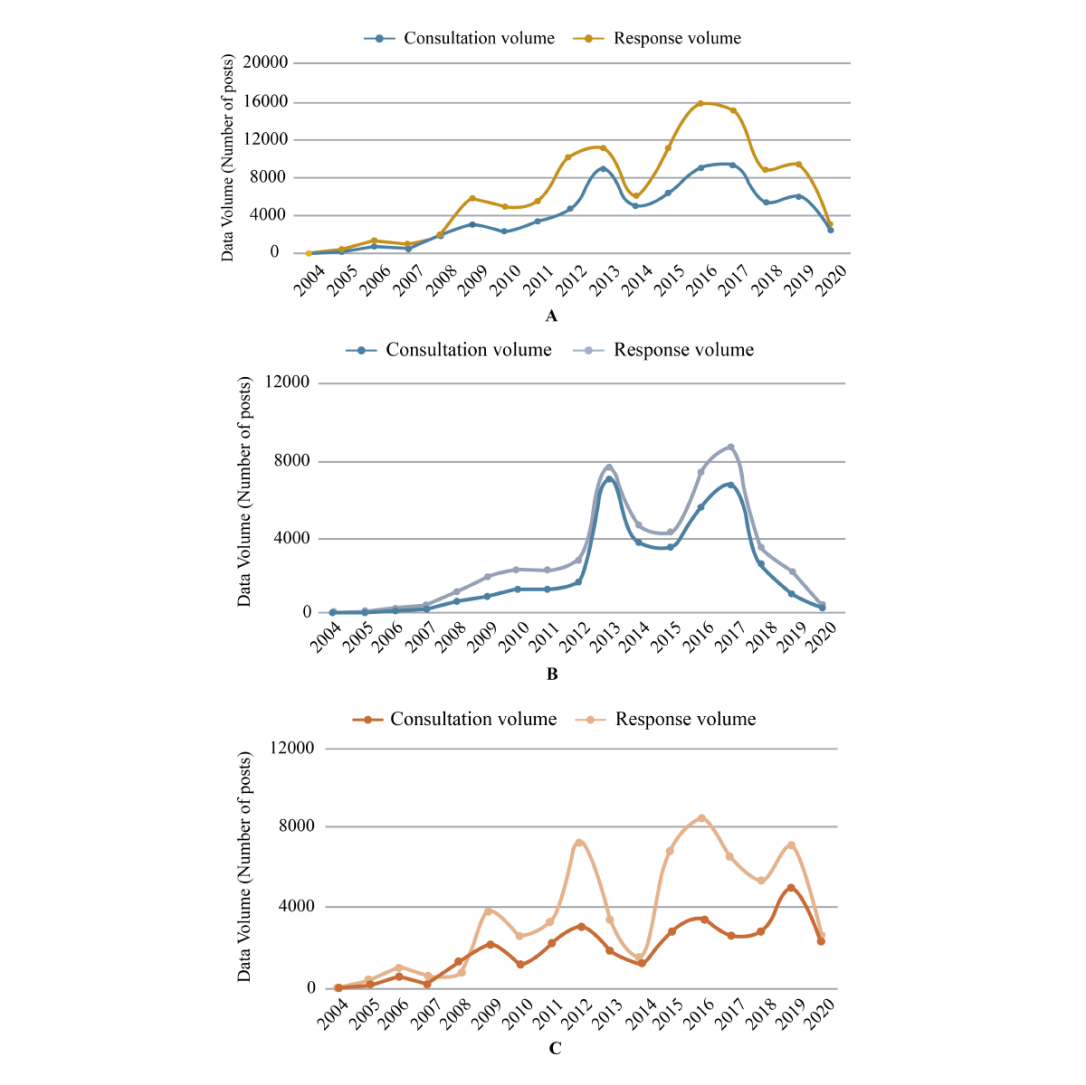
A. time varying curve of post volume in high triglyceride field; B. Change of post volume related to hypertriglyceridemia in Q&A platforms; C. Change of post volume related to hypertriglyceridemia in online consultation platform.

Among the 20 data sources (6 Q&A and 14 online consultation platforms), a total of 69,845 consultation data and 11,1763 response data (61,677 online inquiry platform responses and 50,086 Q&A platform responses) were included for analysis. The distribution of consultations was similar between Q&A platforms (53%) and online consultation platforms (47%). Further, an increase in consultation volume was observed annually up to 2017 and there was an annual decrease in the volume from 2018 to 2020 (Figure 1A) in the combined data set of Q&A platforms and online consultation platforms. The response rates in online consultation and Q&A platforms were 1.88 and 1.34 responses per query, respectively (Figure 1B and 1C). Analysis of keywords through the cloud chart revealed that “treatment’’ and “diet’’ were the most frequently used query and response words in both Q&A and online consultation platforms.

### Patient Distribution and Demographics

Personal data (eg, gender, pregnancy status, relationship to the patient) from a total of 13,155 individuals were obtained by identifying the attributes of the individuals querying and responding on online consultation and Q&A platforms, including 7495/13155 (56.97%) who were identified as male and 5660/13155 (43.03%) who were identified as female. Further, 27.6% of female participants and 20.8% of male participants were consulting on behalf of their parents, spouses, or relatives rather than for themselves. Furthermore, 895/5660 (15.8%) of the female patients had an elevated TG level during pregnancy. From the collected data, age could be recognized in 10,696 records and the age range of 21-60 years accounted for more than 85% of the patients. Young and middle-aged (21-60 years) individuals were the main population utilizing online consultation and Q&A platforms for hypertriglyceridemia. A total of 36,294 records clearly included detection of the TG level. Among these, 73.3% (26603/36294) of the patients had reported only high TG levels and 16.7% (9691/36294) of patients had both elevated TG and cholesterol levels. Among these two groups, the proportion of patients with mild to moderately elevated TG levels was similar (Table 5). Further, 58.0% (94/162) of patients had elevated TG and cholesterol (mild to moderate) levels during pregnancy. Among patients with only high TG, 34% (83/244) had severe elevated TG levels (Table 5).

**Table 5:** Distribution of the type of hypertriglyceridemia among the general population (N=36,294) and pregnant population (n=895) using online platforms.

Type of hypertriglyceridemia		Patients, n (%)
**Elevated triglycerides alone (n=26,603)**		
	Edge elevation	4788 (18.0)
	Mild to moderate elevation	13,994 (52.6)
	Severe elevation	7821 (29.4)
**Combined elevated triglycerides and cholesterol (n=9691)**		
	Edge elevation	2225 (23.0)
	Mild to moderate elevation	4989 (51.5)
	Severe elevation	2477 (25.6)
**Pregnant patients with elevated triglycerides alone (n=244)**		
	Edge elevation	27 (11.1)
	Mild to moderate elevation	134 (54.9)
	Severe elevation	83 (34.0)
**Pregnant patients with elevated triglyceride and cholesterol (n=162)**		
	Edge elevation	25 (15.4)
	Mild to moderate elevation	94 (58.0)
	Severe elevation	43 (26.5)
**Total (N=36,294)**		
	Edge elevation	7013 (19.3)
	Mild to moderate elevation	18,983 (52.3)
	Severe elevation	10,298 (28.4)

A total of 8888 patients complained of symptoms related to hypertriglyceridemia, accounting for 12.7% of the total consultation data. Among these, the most frequently mentioned location of symptoms was the head (n=4478), followed by the chest (n=1919). The typical symptoms of the head included dizziness and headache, while the symptoms of the chest mainly included chest tightness, chest pain, palpitation, and shortness of breath. Further, a total of 1745 patients reporting secondary diseases were identified, including 1.9% reporting atherosclerotic cardiovascular disease (ASCVD) and 0.6% reporting hypertriglyceridemia-induced pancreatitis. Among the patients over 60 years old (n=1027), the incidence of self-reported ASCVD and hypertriglyceridemia-induced pancreatitis was 9.6% and 0.3%, respectively, suggesting that the incidence of ASCVD increases with age. Moreover, the incidence of central vascular disease (coronary heart disease/myocardial infarction) was higher than that of cerebrovascular disease (cerebral infarction) and the incidence of cerebral infarction in patients over 60 years old was higher than that in patients less than 60 years old.

### Demand Analysis

**Figure 2:**
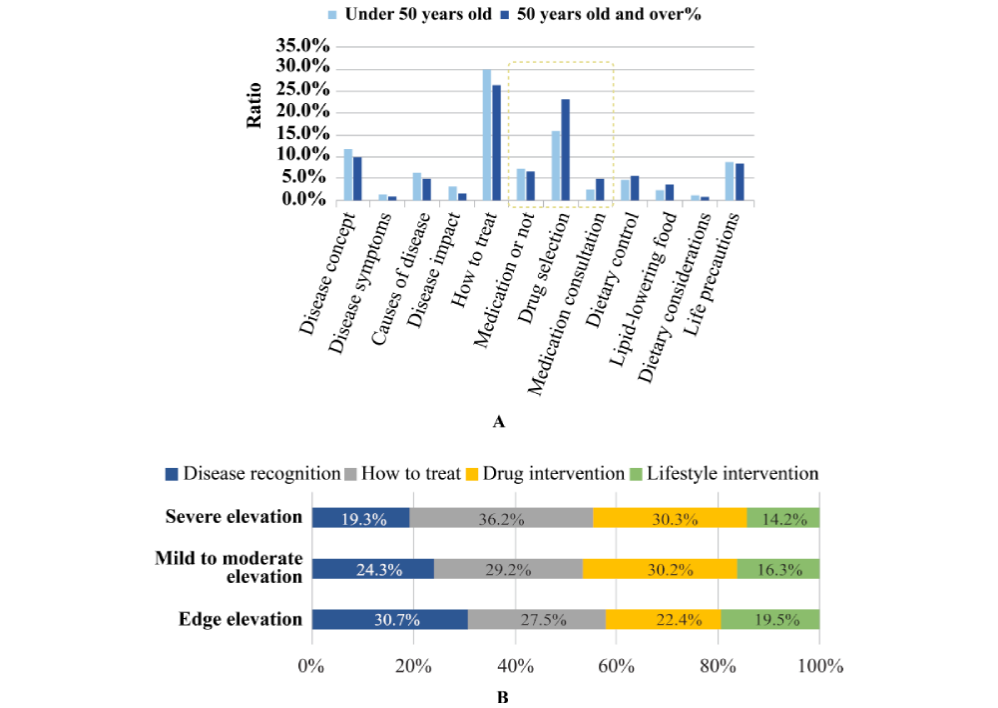
A. Demands of Hypertriglyceridemia patients on the internet based on age (under 50 years old vs 50 years old and over); B. Comparison of demands of different types of hypertriglyceridemia patients on the Internet.

The top 10 demands of patients with hypertriglyceridemia using the online consultation and Q&A platforms are presented in Table 6. Among these consultations, 12,321 were related to disease perception and 37,599 were related to intervention/treatment. “How to treat” was the top-ranked demand of patients with hypertriglyceridemia on the online platforms, followed by drug selection and disease concept. Further analysis based on age groups demonstrated that demand for “disease perception” and “how to treat” was higher in patients under 50 years old (n=6941; 24.2% and 30.1%, respectively) in comparison to that of patients over 50 years old (n=3755; 18.4% and 26.6%, respectively). By contrast, a higher proportion of patients aged >50 years had a demand for drug intervention counseling (35.6% vs 27.0%), whereas there was no significant difference between the two age groups in terms of counseling for lifestyle interventions (19.4% vs 18.7%) (Figure 2A). Furthermore, demand analysis based on the level of TG elevation demonstrated that disease cognition counseling (30.7%) was the highest demand for patients with marginal elevation of TG levels, whereas patients with mild to moderate and severe TG elevation paid more attention to drug intervention (30.2% and 30.3%, respectively) (Figure 2B).

**Table 6:** Top 10 demands of patients with hypertriglyceridemia on the internet.

Priority rank	Patient request	Consultation volume	Category
1	How to treat (how to treat/how to do)	14,194	How to intervene
2	Drug selection (what drug/what drug to take)	9782	Drug intervention
3	Disease concepts (what are triglycerides/what is hyperlipidemia)	6281	Disease perception
4	Lifestyle precautions (What to pay attention to in daily life)	3789	Lifestyle interventions
5	Disease causes (what causes/is it hereditary)	3477	Disease cognition
6	Medication (need medication)	3042	Medication intervention
7	Dietary control (how to control your diet/what you can’t eat),	2551	Lifestyle interventions
8	Lipid-lowering foods (what foods to eat to reduce blood lipids)	1994	Lifestyle interventions
9	Disease impact (what hazards/causes)	1973	Disease cognition
10	Diagnosis and treatment criteria (how serious a high lipid/TG^a^ level)	1814	Disease recognition

^a^TG: triglyceride.

Overall, the composition of patients’ demands on the two types of platforms was compared by including 25,561 data from Q&A platforms and 24,359 data from online consultation platforms. The proportion of consultations related to disease perception on Q&A platforms was higher than that on online consultation platforms, while the proportion of consultations related to drug interventions on online consultation platforms was higher, especially drug selection (22.9% vs 16.4%) and medication consultation (4.9% vs 1.3%), in comparison to that on Q&A platforms. With respect to lifestyle interventions, the proportion of demands related to diet control on the online consultation platforms was higher than that on the Q&A platforms (5.3% vs 4.9%). On the whole, patients from online consultation platforms tend to consult on more specific intervention measures (34.5%), whereas on online Q&A platforms, patients tend to ask broad questions such as those related to disease concepts (29%) and lifestyle precautions.

### Interventions

Table 7 provides an overview of the lifestyle interventions in patients with hypertriglyceridemia using online platforms. The data suggested that lifestyle intervention measures mainly involve diet control. Further, the choice of existing intervention measures reflects the patient’s perception on intervention measures to a certain extent. Compared with the restriction of fat intake and appropriate exercise, the knowledge of other lifestyle interventions was inadequate. In terms of drug interventions, 6746 patients actively mentioned the existing interventions with the most frequent drug interventions including fibrates, followed by statins and traditional Chinese lipid-lowering drugs (Table 7). Further, 275 patients reported the use of a combination of drugs; among these, 85.8% (n=236) and 14.2% (n=39) indicated the use of a combination of two and three drugs, respectively. Furthermore, the most relevant factor for the selection of drug interventions was safety, followed by the efficacy of drugs (Table 7).

**Table 7:** Online consultations about lifestyle and drug interventions, and factors related to drug selection in patients with hypertriglyceridemia on the internet.

Intervention or factor		Patients, n (%)
**Lifestyle intervention choice (n=1259)**		
	Smoking cessation	130 (10.3)
	Weight control	155 (12.3)
	Appropriate exercise	414 (32.8)
	Limit carbohydrates	96 (7.6)
	Limit alcohol consumption	148 (11.8)
	Limit oil use	277 (22.0)
	Dietary control	657 (52.2)
**Drug intervention choice (n=6746)**		
	Drug combination	275 (4.1)
	Niacin	146 (2.2)
	Traditional Chinese medicine	1214 (18.0)
	Statins	2889 (42.8)
	Fibrates	3327 (49.3)
	Other agents	148 (2.2)
**Factors influencing drug selection (n=2704)**		
	Economics	30 (1.1)
	Brand	31 (1.1)
	Traditional Chinese medicine	470 (17.4)
	Effectiveness	1190 (44.0)
	Safety	1393 (51.5)

### Response Suggestion Analysis

#### Overview of Response Data

A total of 38,101 data were related to hospital information of doctors, 35,429 data were related to regional information, and 35,057 data were related to hospital-level information. The doctors from the Shandong, Hebei, and Liaoning provinces had the highest activity, while doctors from other inland areas such as the Henan, Jiangxi, and Jilin provinces had a similar voice volume on online consultation platforms. Further, among respondents, the highest proportion of doctors with professional titles were attending doctors (37.1%) and residents (21.5%), whereas associate chief and chief physicians accounted for 18.1% and 12.2%, respectively, along with a certain proportion of nurses (10.8%) and other health care professionals, including pharmacists, technicians, and inspectors.

#### Response Analysis From Online Consultation Platforms

**Figure 3:**
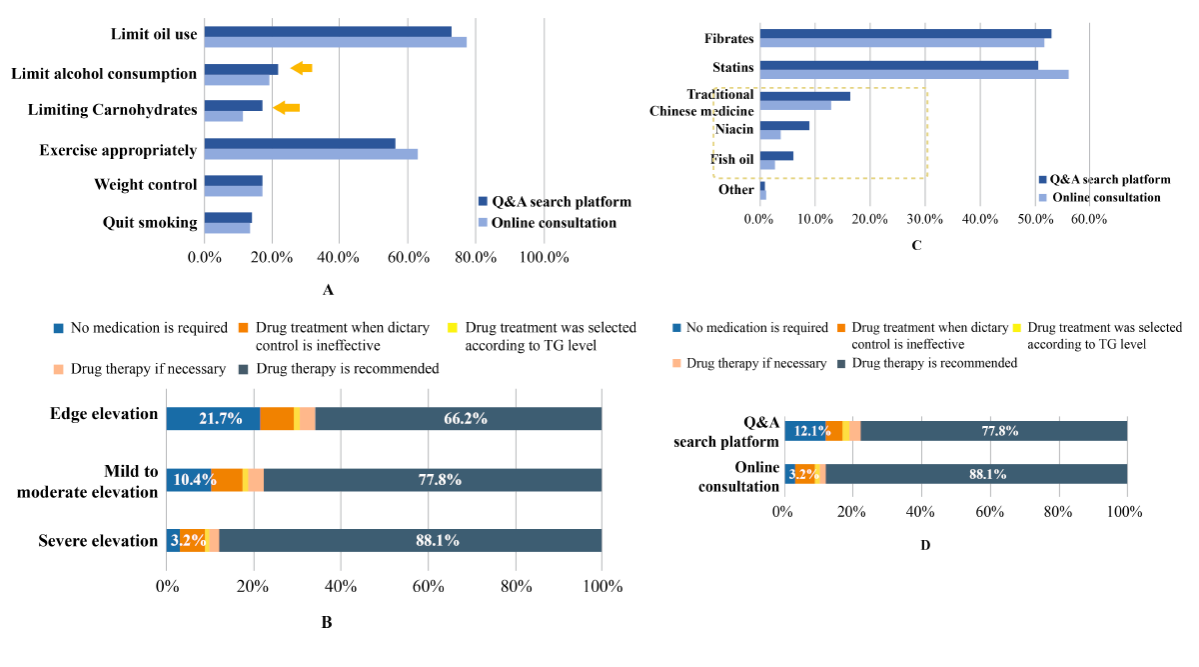
A: Frequency of suggestions for lifestyle intervention on Q&A search and online consultation platforms; B: Composition of medication suggestions for different types of patients based on TG levels on the online consultation platform (n=17243); C: Frequency of recommended drugs Q&A search and online consultation platforms; D: Recommendations on medication for patients with severe triglyceride elevation on Q&A search platform.

A total of 43,188 data were considered for analyzing the integrity of lifestyle intervention suggestions on online platforms. Among these, reduce fats (77.2%) and appropriate exercise (63.0%) were the most frequent responses, while the proportions of explicit mentions of other potential interventions were low (<20%). In addition, 12.4% (n=5357) of the responses suggested “diet control” without a specific explanation (Figure 3A).

Further, there were 36,851 responses for patients who provided specific TG test values, resulting in 17,243 suggestions for medications. The data indicated that drug recommendations on the online consultation platforms were related to the level of TG, wherein a higher proportion of drug recommendations was provided for patients with severe hypertriglyceridemia (88.1%), followed by those for patients with mild to moderate (77.8%) and marginally elevated (66.2%) TG levels, respectively (Figure 3B). However, the proportion of drug recommendations remained high among all categories of patients. Furthermore, on the online consultation platforms, there were 24,826 responses for patients who did not provide specific TG test values, among which 9109 (36.7%) responses provided medication suggestions. In terms of drug recommendations on the online consultation platforms, statins (56.1%) and fibrates (51.7%) were the most common drugs recommended in the doctors’ replies, followed by traditional Chinese medicine lipid-lowering drugs (12.8%) such as Xuezhikang, Jiangzhining, and Jiangzhi tablets; Hedan tablets; and others (Figure 3C).

#### Response Analysis From Q&A Search Platforms

A total of 32,458 data were retrieved for analyzing the integrity of Q&A search platforms for lifestyle interventions. The average word count in the response data of Q&A search platforms was 186.1 and that of online consultation platforms was 131.9. Furthermore, the proportion of recommendations for carbohydrate restriction (17.1% vs 11.3%) and alcohol restriction (21.8% vs 19.0%) was higher on the Q&A search platforms than on the online consultation platforms, indicating that the integrity of lifestyle intervention suggestions of Q&A search platforms was higher than that of online consultation platforms (Figure 3D). In case of drug intervention recommendations, the proportion of patients with drug recommendations for those with severe hypertriglyceridemia was lower in Q&A search platforms when compared to that of online consultation platforms (77.8% vs 88.1%) (Figure 3D). Although statins and fibrates were the most popular recommendations in Q&A search platforms, lipid-lowering drugs such as traditional Chinese medicine, nicotinic acid, and fish oil were also frequently mentioned as the recommended drugs (Figure 3C).

Moreover, the proportion of supplements recommended by Q&A search platforms was slightly higher when compared to that on online consultation platforms (3.1% vs 1.6%), and health products other than fish oil were also more frequently recommended on Q&A platforms than on online consultation platforms (56.0% vs 45.8%). In addition, the proportion of recommendations for a lipid-lowering diet for patients was higher on Q&A search platforms than on online consultation platforms (13.7% vs 7.5%). There was considerable overlap in supplement recommendations between Q&A and online consultation platforms.

## Discussion

### Principal Findings

The use of digital technology has increased exponentially in recent years, which is now frequently utilized in health care systems [6]. At this juncture, SML is an untapped approach that can provide a window into patients’ choices and experiences [10]. In particular, social media platforms, including peer-to-peer communication and online consultation platforms, are easily accessible for addressing patients’ educational needs on specific health conditions and providing real-time interactions with health care professionals for support and management [36].

The results of this study demonstrated that out of 20 data sources, a notable number of consultations (n=69,845) and corresponding responses (n=1,11,763) were obtained, indicating the demand of digital platforms in the health care system, specifically for hypertriglyceridemia. Notably, on the online consultation platforms, doctors from inland areas have relatively high voice volumes and exert a great influence. Moreover, patients with hypertriglyceridemia using online consultation platforms tended to consult more on intervention measures, whereas those using Q&A platforms tended to ask disease perception– and life precautions–related questions. In addition, many of the patients’ questions were not specific and clear, which indicates that the average perception level of patients about the disease and intervention measures is not high. Moreover, the lifestyle intervention measures adopted by patients mainly include limiting fat intake and proper exercise, which indicates the weak perception of patients on other lifestyle interventions such as abstinence of alcohol, limiting carbohydrate intake, controlling weight, and smoking cessation. Likewise, compared with the older patients, young patients appear to pay more attention to disease perception and treatment along with clinical manifestations, disease progress, and the long-term impact on the body and life. However, 41.6% of patients aged 50 years and above consulted on whether to use drugs, drug selection, medication consultation, and diet control, suggesting that they may have a better understanding of the disease and the corresponding intervention measures. Meanwhile, patients with severely elevated TG levels using the online platforms paid more attention to the safety of long-term drug use than to other factors, including drug efficacy. Additionally, these patients have a clear intention to use drug intervention and few patients have a strong willingness for the use of traditional Chinese medicine.

In terms of response suggestions, the proportion of drug intervention recommendations based on the TG level was higher in online consultations, whereas the proportions of lifestyle intervention and supplement recommendations were higher on Q&A platforms, representing the integrity and uniqueness of each platform. On the online consultation platforms, doctors tended to recommend medication for all patients, accounting for 65%-85% of the recommendations, which may lead to unnecessary medication use. Consequently, the integrity of internet doctors in recommending accurate lifestyle and drug interventions still needs to be improved.

Although SML is a relatively new approach, its main strength is that it provides insights by offering listening and learning from online conversations of patients without any research or patient requirement burden. In addition, such insights offer fresh and more unbiased perspectives compared to those obtained with conventional approaches [7]. The findings from SML analyses may offer preliminary evidence prior to conventional qualitative research and can aid in the design of subsequent survey questionnaires that will pave the way for exploring patients’ insights and treatment patterns in detail [37]. Cook et al [7] utilized SML to obtain patients’ perspectives on symptoms, diagnosis, and comorbidities associated with chronic obstructive pulmonary disease and its impact on patients’ quality of life. They reported that shortness of breath, cough, and mucus production would be the most desirable aspects of disease management from a patient’s perspective [7]. Subsequently, Booth et al [10] conducted a disease-specific SML study in patients with acute myeloid leukemia to uncover treatment experiences, highlighting the existence of an information gap between patients and caregivers in terms of treatment options that aided clinicians to make better-informed recommendations for their patients [10]. In another study, Cook et al [9] evaluated the patient experience with different indications for dry-eye disease through an SML approach using natural language processing and the results obtained from this study contributed to effectively understanding patient experiences and their unmet knowledge needs.

### Strengths and Limitations

To the best of our knowledge, this is the first SML study evaluating the disease cognition level and choice of intervention measures among patients with hypertriglyceridemia using online platforms, specifically in the Chinese population. Additionally, the study was novel as it involves the use of unsolicited, unstructured social media data (including Q&A platforms and online consultation platforms) related to hypertriglyceridemia using big data analysis and artificial intelligence.

This study also has several limitations. First, the users of online platforms typically post using screen names, making it difficult for verification of the patients’ identities. Consequently, it is challenging to ascertain if the diagnosis is clinically confirmed or self-diagnosed unless there are clear details describing the hypertriglyceridemia, including laboratory results. Second, owing to ethical and privacy considerations, the relation to the data was not made at the individual level. Third, the analysis was based on the declarations by the online platform users and therefore the symptoms stated may not truly reflect hypertriglyceridemia in spite of our rigorous inclusion criteria and manual curation by health professionals. Finally, there was no follow-up in the analysis of SML as compared with traditional methods or interviews. We acknowledge the potential for duplication due to multiple screen names, bias, and lack of demographic information in certain cases even though we implemented both manual and machine-learning curation and processing techniques.

### Conclusion

To conclude, patients with hypertriglyceridemia actively seek information from both Q&A search and online consultation platforms. However, the integrity of internet doctors’ suggestions on lifestyle interventions and the accuracy on drug intervention recommendations still need to be improved. Further, a combined prospective, qualitative study with SML is required for added rigor and confirmation of the relevance of the findings.

## Appendix

Multimedia Appendix 1 Schematic illustration of data acquisition path.

Multimedia Appendix 2 Schematic illustration of analysis dimensions. TG: triglycerides.

## Abbreviations

ASCVD: atherosclerotic cardiovascular disease
Q&A: question and answer
SML: social media listening
TG: triglyceride

## Data Availability

The data sets generated during and/or analyzed during this study are available from the corresponding author on reasonable request.
