# Supplementary figures and images for: Evaluation of the Needs and Experiences of Patients with Hypertriglyceridemia: Social Media Listening Infosurveillance Study
Source: J Med Internet Res. 2023 Dec 19;25:e44610. doi: 10.2196/44610 (PMC10762621; doi:10.2196/44610)

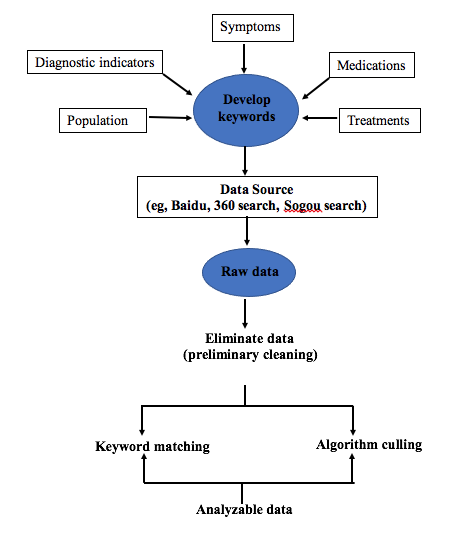

Supplement: Multimedia Appendix 1 [file jmir_v25i1e44610_app1.png]

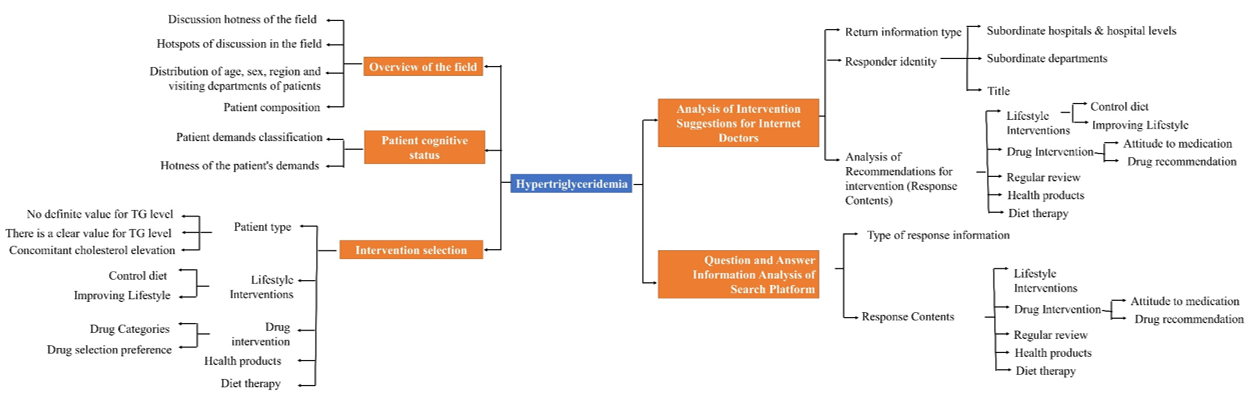

Supplement: Multimedia Appendix 2 [file jmir_v25i1e44610_app2.png]
